# The significance of Hippo pathway protein expression in oral squamous cell carcinoma

**DOI:** 10.3389/fmed.2024.1247625

**Published:** 2024-02-20

**Authors:** Yusuke Amano, Daisuke Matsubara, Atsushi Kihara, Taichiro Yoshimoto, Noriyoshi Fukushima, Hiroshi Nishino, Yoshiyuki Mori, Toshiro Niki

**Affiliations:** ^1^Department of Integrative Pathology, Jichi Medical University, Shimotsuke, Japan; ^2^Department of Diagnostic Pathology, University of Tsukuba, Tsukuba, Japan; ^3^Department of Otolaryngology, Jichi Medical University, Shimotsuke, Japan; ^4^Department of Dentistry, Oral and Maxillofacial Surgery, Saitama Medical Center Jichi Medical University, Saitama, Japan

**Keywords:** Hippo pathway, MST1/2, YAP1, PRMT, oral squamous cell carcinoma

## Abstract

**Introduction:**

The Hippo pathway consists of mammalian sterile 20-like kinase 1/2 (MST1/2), large tumor suppressor 1/2 (LATS1/2), and yes-associated protein (YAP)1. Herein, we present the first report on the significance of major Hippo pathway protein expression in oral squamous cell carcinoma (OSCC).

**Methods:**

The analyses included oral epithelial dysplasia (OED, *n* = 7), carcinoma *in situ* (CIS, *n* = 14), and oral squamous cell carcinoma (OSCC, *n* = 109).

**Results:**

Cytoplasmic expression of MST1, LATS1, and LATS2 was low in OED, CIS, and OSCC. The cytoplasmic expression of MST2 was high in OED (5/7 cases), CIS (9/14 cases), and poorly differentiated OSCC (8/8 cases) but was low/lost in a proportion of differentiated OSCC (60/101 cases). The expression of YAP1 was associated with differentiation; low YAP expression was significantly more frequent in well-differentiated OSCC (35/71 cases), compared to moderately and poorly differentiated OSCC (11/38 cases). An infiltrative invasion pattern was associated with a high expression of MST2 and high expression of YAP1. The high expression of YAP1 was associated with features of epithelial-to-mesenchymal transition (EMT), such as the loss of E-cadherin and high expression of vimentin, laminin 5, and Slug. High expression of protein arginine methyltransferase (PRMT) 1 or 5, which positively regulates YAP activity, was associated with the high expression of YAP1 (*p* < 0.0001).

**Conclusion:**

Among the major Hippo pathway proteins, MST2 displayed a distinctive expression pattern in a significant proportion of differentiated OSCC, suggesting a possible differential role for MST2 depending on the course of OSCC progression. A high YAP1 expression may indicate aggressive OSCC with EMT via PRMTs at the invasive front.

## Introduction

Cancers of the lip and oral cavity are the sixteenth most common neoplasm worldwide, with approximately 378,000 new cases and178,000 deaths in 2020 (1). More than 90% of oral cavity cancer cases are histologically classified as squamous cell carcinoma (SCC) (2). Although advances have been made in the treatment of oral SCC (OSCC), including surgical resection, chemotherapy, and radiotherapy, the 5 years overall survival (OS) rate of OSCC ranges between 24.5% and 50% (3). Comprehensive molecular analyses of head and neck cancers, including OSCC, have identified the amplification of representative target genes, including epidermal growth factor receptor (15%), fibroblast growth factor receptor 1 (10%), and human epidermal growth factor receptor 2 (5%) (4). Therefore, new cancer therapies based on the molecular mechanisms underlying OSCC are required.

The Hippo pathway consists of a large network of proteins that control the end-organ sizes of different tissues by regulating proliferation, cell growth, and apoptosis (5). This pathway comprises a core kinase cascade, starting with the activation of a pair of serine/threonine kinases, mammalian STE20-like protein kinases (MST1/2), which, in turn, activate another pair of kinases, large tumor suppressor kinases (LATS1/2) (5). LATS1/2 phosphorylates the transcriptional activator yes-associated protein 1 (YAP), inducing its transport from the nucleus to cytoplasm (5). When the Hippo pathway is active, YAP1 is phosphorylated by LATS1/2, resulting in interaction with 14-3-3 and cytoplasmic retention, which also leads to polyubiquitination and degradation of YAP/TAZ (5).

When the Hippo pathway is inactive, YAP1 is present in the nucleus and interacts with transcription factors such as TEAD 1–4. The localization and phosphorylation of YAP1 are often used as measures of Hippo pathway activity (5). Previous studies have reported the overexpression of YAP1 in human cancer and revealed a relationship between higher expression or activity of YAP1 and worse patient prognoses in various tumor entities (6). Furthermore, YAP1 has been shown to drive cancer metastasis (6). In OSCC, the Hippo pathway plays an essential role in disease progression and YAP1 activation, both of which correlate with unfavorable prognosis (7, 8).

Protein arginine methyltransferases (PRMT) transfer methyl groups to guanidine nitrogen molecules of arginine residues on their target proteins, which affects gene expression, signal transduction, RNA splicing, and other cellular functions (9).

Recent studies have demonstrated that PRMT 1, a type I PRMT, functions as a positive regulator of YAP1 activity in chondrosarcoma (10), while PRMT5, a type II PRMT, promotes laryngeal cancer cell growth and its invasive capacity through the LATS2 and YAP1 signaling pathways (11).

Although the expression of the major Hippo pathway proteins has been reported in pancreatic cancer (12), their involvement in OSCC remains unclear. In the present study, we examined the expression of major proteins (MST1, MST2, LATS1, LATS2, and YAP1) in the Hippo pathway in OSCC, carcinoma *in situ* (CIS), and its precursor lesion, oral epithelial dysplasia (OED), and investigated the clinicopathological significance of Hippo pathway protein expression in OSCC.

## Materials and methods

### Patients and tumors

Tumor specimens were obtained from 130 patients who underwent surgery for OSCC without neoadjuvant chemoradiotherapy at the Jichi Medical University Hospital between 2010 and 2015. These cases were derived from our previous study cohort (141 cases) (13). Eleven cases were excluded because the tissue blocks were not eligible for further sectioning. The patients had OED (corresponding to moderate dysplasia) (*n* = 7), CIS (*n* = 14), or OSCC (*n* = 109). All histological diagnoses were made according to the 2005 World Health Organization 2005 criteria (14). Information was collected on the following factors for each case: age, sex, primary site, histological grade, tumor stage, lymph node metastasis, Yamamoto–Kohama (YK) classification (15), lymphovascular invasion, perineural invasion, and patient survival. The patient cohort consisted of 70 males and 60 females, ranging in age from 26 to 89 years (mean age: 63.9 years). The primary sites were the tongue (*n* = 95), gingiva (*n* = 18), buccal mucosa (*n* = 6), and the oral floor (*n* = 11). Pathological stage was defined according to the TNM classification established by the American Joint Committee on Cancer and the International Union Against Cancer (16), and cut-off values for the depth of invasion (DOI) and tumor thickness were also based on this classification (16). The follow-up period ranged from 81 to 3,441 days (median, 1,520 days).

The morphological pattern of invasion was selected according to the YK classification as follows: grade 1, well-defined borderline; grade 2, less prominent borderline; grade 3, a group of cells with no distinct borderline; grade 4C, diffuse invasion of the cord-like type; grade 4D, diffuse invasion of the widespread type (15).

The study protocol was approved by the Ethics Committee of the Jichi Medical University (approval ID: A15-269).

### Immunohistochemistry

Specimens for immunohistochemical evaluation were collected from representative areas of the tumors. Immunohistochemistry was performed using primary antibodies against the following antigens: MST1, MST2, LST1, LST2, YAP1, E-cadherin, vimentin, Slug, laminin 5, PRMT1, and PRMT5. The details of the antibodies used, antigen retrieval methods, and antibody dilutions are listed in Supplementary Table S1.

The immunoperoxidase polymer method (Histofine Simple Stain MAX PO, Nichirei, Tokyo, Japan) was used to detect all primary antibodies. 3,3′-Diaminobenzidine tetrahydrochloride was used as a chromogen, and hematoxylin was used as a light counterstain.

### Evaluation of immunohistochemical findings

Immunohistochemical staining was independently analyzed by two pathologists (YA and DM) who were blinded to the clinical data obtained through microscopic examinations. LATS1, LATS2, and YAP1 immunoreactivity was evaluated as previously described (17–19).

The intensity of MST1 and MST2 staining was graded as negative (0), mild (1), moderate (2), and strong (3). The percentage of positive cells was divided into four categories: (0), no immunostaining; (1), <10% reactive cells; (2), 11%–50% reactive cells; and (3), >50% reactive cells. In the statistical analyses, these categories were added and further classified as low (0, 1, 2, and 3) or high (4, 5, and 6), as reported by Drexler et al. (12), with slight modifications.

Immunoreactivities for Vimentin, PRMT1, PRMT5, E-cadherin, Slug, and laminin 5 were evaluated using previously described methods (13).

### Statistical analyses

The chi-squared test was used to examine the relationships between Hippo pathway protein expression, clinicopathological factors, epithelial-to-mesenchymal transition (EMT) marker expression, and PRMT expression.

The relapse-free survival (RFS) and OS rates were calculated according to the Kaplan–Meier method, and differences between groups were assessed using the log-rank test. Univariate and multivariate analyses were performed using the Cox proportional hazards model [described as hazard ratios with 95% confidence intervals (95% CIs), together with *p*-values]. Statistical significance was set at *p* < 0.05. All statistical analyses were performed using the statistical software Bell Curve for Excel (Social Survey Research Information Co., Ltd.).

## Results

### Expression of MST1 and MST2 in the normal squamous epithelium, OED, CIS, and SCC

**Figure 1:**
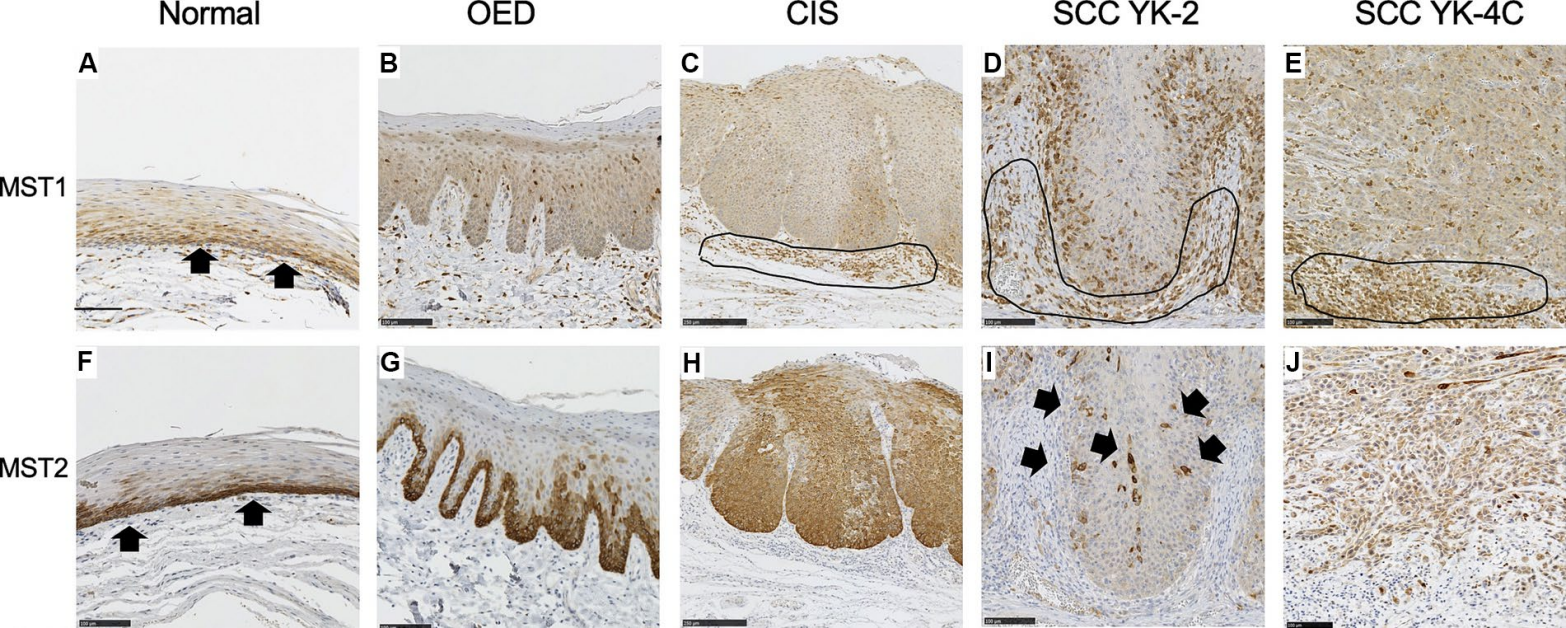
Expression of MST1 and MST2 in the normal epithelium, oral epithelial dysplasia (OED), carcinoma *in situ* (CIS), and squamous cell carcinoma (SCC). **(A)** Cytoplasmic expression of MST1 was weakly observed in the basal and parabasal cell layers of the normal epithelium (arrows). **(B)** Cytoplasmic expression of MST1 was weakly observed in dysplastic cells that occupy the basal 2/3 of the epithelium. **(C)** Cytoplasmic expression of MST1 in neoplastic cells. **(D)** Cytoplasmic expression was observed MST1 in YK-2, OSCC. **(E)** Cytoplasmic expression of MST1 in YK-4C. **(F)** Cytoplasmic expression of MST2 in the basal and parabasal cell layers of the normal epithelium. The MST2 expression was especially high in the basal layer (arrows). **(G)** Cytoplasmic expression of MST2 in dysplastic cells that occupy the basal 2/3 of the epithelium. In 5 of 7 OED cases, the MST2 expression was especially high in dysplastic cells in the basal layer. **(H)** Diffuse high cytoplasmic expression of MST2 was observed in neoplastic cells in the majority of CIS (9 of 14 cases). **(I)** The MST2 expression was largely lost in neoplastic cells of YK-2 OSCC, with a few MST-positive cells remaining (arrows). This pattern was observed in the large majority of YK-1–3 cases (28 of 40 cases). **(J)** Diffusely high cytoplasmic expression of MST2 in neoplastic cells of YK-4C OSCC. Such high expression of MST2 was observed in 37 of 69 YK-4C or 4D cases, with statistical difference as compared to YK-1–3 cases (*p* < 0.0169). Note that inflammatory cells in the stroma were also stained positive for MST1 in **(C–E)** (encircled). Bar: 100 μm.

We examined the MST1 staining patterns in the normal epithelium (the unaffected area of the tongue in OED cases), OED, and CIS. The cytoplasm of the basal cells of the normal squamous epithelium was weakly positive for MST1 (Figure 1A). In all OED cases, uniformly positive MST1 staining was detected in the cytoplasm of the dysplastic cells (Figure 1B). In all CIS and SCC cases, uniformly positive MST1 staining was detected in the cytoplasm of the neoplastic cells (Figures 1C–E). The intensity of MST1 staining did not significantly differ among normal epithelium, OED, CIS, SCC, and clinicopathological factors.

The cytoplasm of the basal cells of the normal squamous epithelium was positive for MST2 (Figure 1F). In five OED cases, the cytoplasm of the dysplastic cells was strongly positive for MST2 (Figure 1G). In the remaining two cases of OED, uniformly positive MST2 staining was weakly detected in the cytoplasm of dysplastic cells (Table 1). In nine CIS cases, the cytoplasm of the dysplastic cells was strongly positive for MST2 (Figure 1H). In the remaining five cases of CIS, uniformly positive MST2 staining was weakly detected in the cytoplasm of the dysplastic cells (Table 1). MST2 was differentially expressed in SCC, depending on tumor differentiation and mode of invasion. In a significant proportion of differentiated SCC (60/101 of grade 1 and 2 cases), MST2 expression was largely low/absent, while it was highly expressed in all poorly differentiated OSCC (Table 1). Similarly, the proportion of MST2 high cases were significantly low in SCC with well-defined invasive pattern (28/40 of YK-1–3 cases) as compared to SCC with a diffuse invasion pattern (32/69 of YK-4C and 4D cases) (Figures 1I,J and Table 1).

**Table 1:** Relationships between the localization of MST2 and clinicopathological factors.

		MST2		
Factors	Total	Low	High	*p*-value
**Age**				
Over 60	84	41	43	0.6362
Under 60	46	26	20	
**Gender**				
Male	70	34	36	0.4647
Female	60	33	27	
**Location**				
Tongue	95	49	46	0.9879
Others	35	18	17	
**Histological type**				
OED	7	2	5	0.0683^*^
CIS	14	5	9	
Grades 1, 2	101	60	41	**0.0011** ^**^
Grade 3	8	0	8	
**pT**				
pT1, 2, 3	93	54	39	0.1267
pT4	16	6	10	
**Stage**				
I, II	77	49	28	**0.0052**
III, IV	32	11	21	
**Lymphovscular invasion**				
Negative	43	26	17	0.3586
Positive	66	34	32	
**Neural invasion**				
Negative	64	45	19	**0.0001**
Positive	45	15	30	
**Lymph node metastasis**				
Negative	68	50	18	**<0.0001**
Positive	41	10	31	
**YK**				
1, 2, 3	40	28	12	**0.0169**
4C, 4D	69	32	37	

Age, gender, and location including OED and CIS. ^*^OED, CIS vs. Grades 1–3. ^**^Grades 1, 2 vs. Grade 3. The chi-squared test was used to evaluate the associations among MST2 expression and clinicopathological parameters. Bold, *p* < 0.05.

The relationship between MST2 expression and other clinicopathological factors of OSCC is shown in Table 1. The intensity of MST2 staining was not associated with age, sex, tumor location, pathological T (pT) stage, DOI, or lymphovascular invasion (Table 1).

### Expression of LATS1 and LATS2 in the normal squamous epithelium, OED, CIS, and SCC

**Figure 2:**
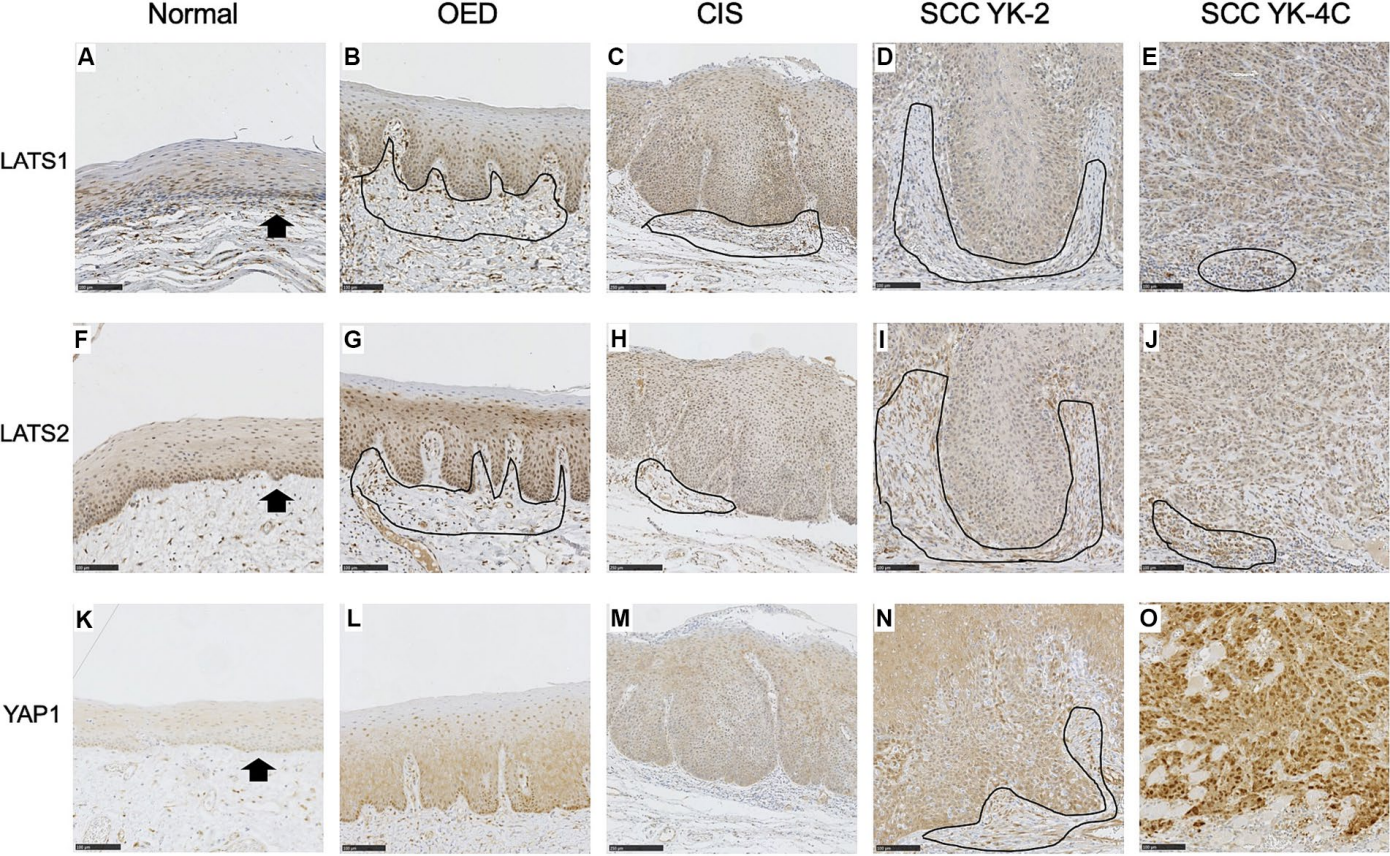
Expression of LATS1, LATS2, and YAP1 in the normal epithelium, oral epithelial dysplasia (OED), carcinoma *in situ* (CIS), and squamous cell carcinoma (SCC). **(A)** Weak expression of LATS1 mainly in the cytoplasm of the basal and parabasal cells of the normal epithelium (arrows). **(B)** Weak expression of LATS1 mainly in the cytoplasm of dysplastic cells of most OED (5 of 7 cases). **(C)** Weak expression of LATS1 mainly in the cytoplasm of neoplastic cells of most CIS (9 of 14 cases). **(D)** Weak expression of LATS 1 mainly in the cytoplasm of neoplastic cells of most YK-2 OSCC (34 of 40 cases). **(E)** Weak expression of LATS1 mainly in the cytoplasm of neoplastic cells of most YK-4C OSCC (60 of 69 cases). **(F)** Weak expression of LATS2 mainly in the cytoplasm of the basal and parabasal cells of the normal epithelium (arrows). **(G)** Weak expression of LATS2 mainly in the cytoplasm of dysplastic cells of OED (4 of 7 cases). **(H)** Weak expression of LATS2 mainly in the cytoplasm of neoplastic cells of CIS (9 of 14 cases). **(I)** Weak expression of LATS2 mainly in the cytoplasm of neoplastic cells of most YK-2 OSCC (38 of 40 cases). **(J)** Weak expression of LATS2 mainly in the cytoplasm of most YK-4C OSCC (63 of 69 cases). **(K)** Vary faint cytoplasmic expression of YAP1 in the basal cell layer of the normal epithelium. **(L)** Weak expression of YAP1 mainly in the cytoplasm of dysplastic cells of OED (6 of 7 cases). **(M)** Weak expression of YAP1 mainly in the cytoplasm of neoplastic cells of most CIS (12 of 14 cases). **(N)** Weak expression of YAP1 mainly in the cytoplasm of neoplastic cells of most YK-2 OSCC (32 of 40 cases). **(O)** High expression of YAP1 in both nuclei and cytoplasms of neoplastic cells of most YK-4C OSCC (55 of 69), with statistical difference as compared to YK-1–3 OSCC (8 of 40) (<0.0001). Note that stromal cells (mainly inflammatory cells and fibroblasts) were also stained positive for LATS1 **(B–D)**, LATS2 **(G–J)**, and YAP1 **(N)** (encircled). Bar: 100 μm.

The cytoplasm of basal cells of normal squamous epithelium was weakly positive for LATS1 and LATS2 (Figures 2A,F). Staining was mainly cytoplasmic. In most OED, CIS, and SCC cases (5/7 OED, 9/14 CIS, and 94/109 SCC), uniformly positive LATS1 staining was weakly detected, mainly in the cytoplasm of dysplastic or neoplastic cells (Figures 2B–E).

In the remaining cases of OED, CIS, and SCC, positive LATS1 staining was strongly detected, mainly in the cytoplasm of dysplastic or neoplastic cells (Supplementary Table S2).

In most OED, CIS, and SCC cases (4/7 OED, 9/14 CIS, and 101/109 SCC), uniformly positive LATS2 staining was weakly detected, mainly in the cytoplasm of dysplastic or neoplastic cells (Figures 2G–J).

In the remaining cases of OED, CIS, and SCC, positive LATS2 staining was strongly detected, mainly in the cytoplasm of dysplastic or neoplastic cells (Supplementary Table S3).

The intensity of LATS1 staining correlated with intraepithelial lesions (OED and CIS vs. OSCC) and low-grade lesions (grades 1 and 2 vs. grade 3) (Supplementary Table S2, *p* = 0.0285 and 0.0429, respectively). The intensity of LATS2 staining correlated with intraepithelial lesions (OED and CIS vs. OSCC) and was marginally associated with early stages (I, II vs. III, IV) (Supplementary Table S3, *p* = 0.0001 and 0.0502, respectively).

### Expression of YAP1 in the normal squamous epithelium, OED, CIS, and SCC

The cytoplasm of the basal cells of the normal squamous epithelium was very weakly positive for YAP1 (Figure 2K). In all OED cases, uniformly positive YAP1 staining was detected in the cytoplasm of the dysplastic cells (Figure 2L). In most CIS and YK-2 SCC cases, uniformly positive YAP1 staining was detected in the cytoplasm of the neoplastic cells (Figures 2M,N). In YK-4C SCC cases, cancer cells often displayed diffuse strong positivity for YAP1 in the nucleus and cytoplasm (Figure 2O). High YAP1 expression was correlated with nuclear and cytoplasmic localization (hereinafter referred to as YAP1 high), while low YAP1 expression was correlated with cytoplasmic localization (hereafter referred to as YAP1 low). These findings are consistent with those of the previous report (20, 21).

The intensity of YAP1 staining did not significantly differ among the normal epithelium, OED, CIS, and YK-1–3 SCC but was stronger in YK-4C and 4D cases than in YK-1–3 cases. The relationship between YAP1 expression and clinicopathological factors of OSCC is shown in Table 2. OED and CIS were associated with YAP1 low (Table 2). YAP1 expression was associated with age (>60 vs. ≤60 years), sex (male vs. female), OSCC histology (grades 1 and 2 vs. grade 3), and YK (1, 2, 3 vs. 4C, 4D) (*p* = 0.0148, 0.0055, 0.012, and <0.0001, respectively).

**Table 2:** Relationships between YAP1 localization and clinicopathological factors.

		YAP1		
Factors	Total	Low	High	*p*-value
**Age**				
Over 60	84	48	36	**0.0148**
Under 60	46	16	30	
**Gender**				
Male	82	48	34	**0.0055**
Female	48	16	32	
**Location**				
Tongue	95	42	53	0.0593
Others	35	22	13	
**Histological type**				
OED	7	6	1	**0.0003** ^*^
CIS	14	12	2	
Grades 1, 2	101	46	55	**0.012** ^**^
Grade 3	8	0	8	
**pT**				
pT1, 2, 3	93	42	51	0.1315
pT4	16	4	12	
**Stage**				
I, II	77	35	42	0.2861
III, IV	32	11	21	
**Lymphovscular invasion**				
Negative	43	20	23	0.4621
Positive	66	26	40	
**Neural invasion**				
Negative	75	36	39	0.0687
Positive	34	10	24	
**Lymph node metastasis**				
Negative	81	37	44	0.2112
Positive	28	9	19	
**YK**				
1, 2, 3	40	32	8	**<0.0001**
4C, 4D	69	14	55	

Age, gender and location including OED and CIS. ^*^OED, CIS vs. Grades 1–3. ^**^Grades 1, 2 vs. Grade 3. The chi-squared test was used to evaluate the associations among YAP1 expression and clinicopathological parameters. Bold, *p* < 0.05.

**Figure 3:**
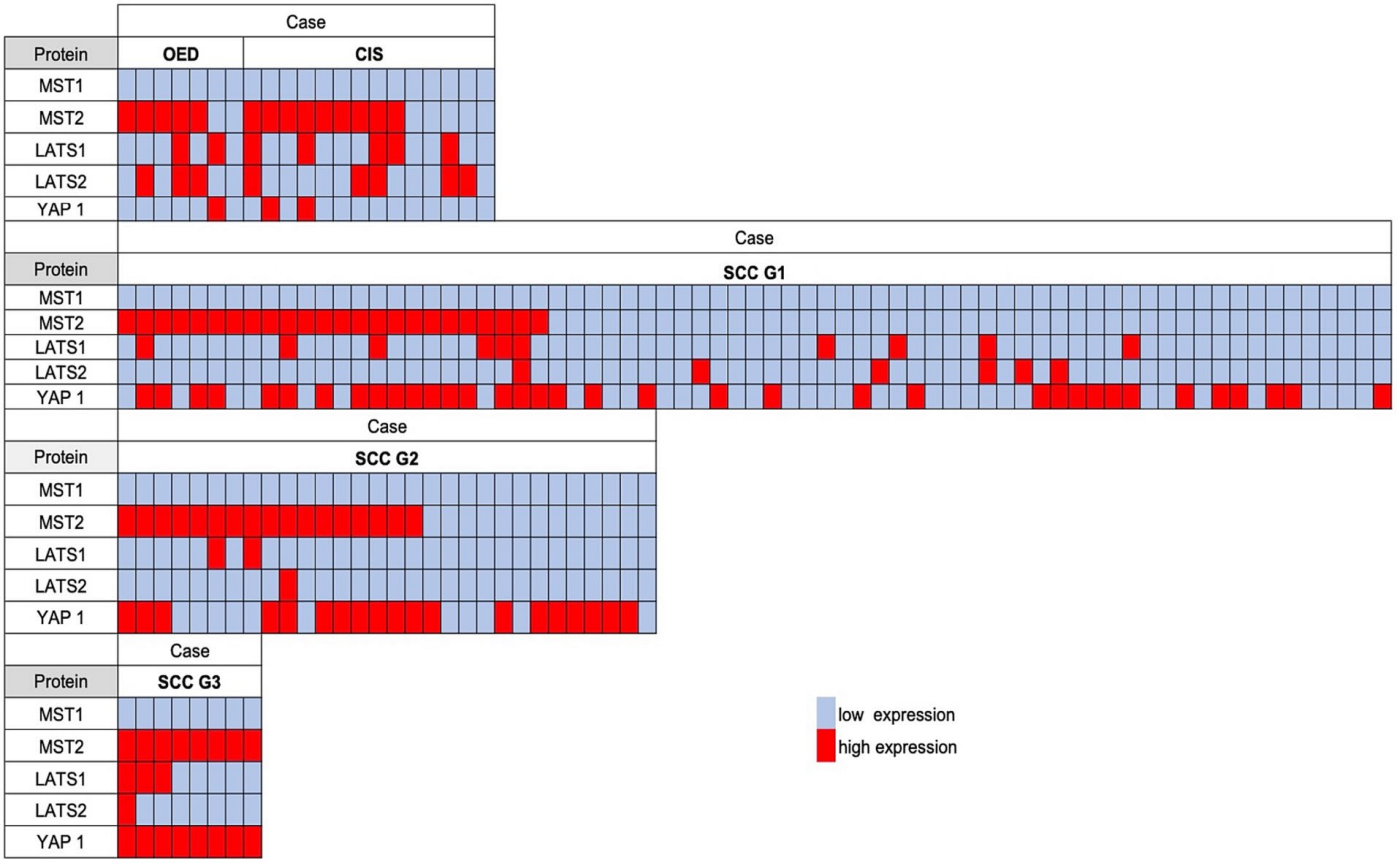
The comparison of the semiquantitative immunohistochemical expression of each Hippo pathway protein in OSCC, carcinoma *in situ* (CIS), and oral epithelial dysplasia (OED). A red-filled box indicates a high expression; an open box indicates a low expression. The concomitant high expression of MST2 and YAP1 was associated with a higher histological grade.

We then compared the significance of Hippo pathway protein expression in OED, CIS, and OSCC to clarify the differences according to the histological grade (Figure 3). High MST2 expression was detected in 71.4% of OED cases (5/7) and 64.3% of CIS cases (9/14). The concomitant high expression of MST2 and YAP1 in OSCC cases was associated with a higher histological grade, that is, in 17/71 grade 1 cases (23.9%), 11/30 grade 2 cases (33.3%), and 8/8 grade 3 cases (100%). The present results suggest that the co-expression pattern of MST2 high and YAP1 is highly correlated with aggressive histologic features of OSCC.

### Relationships among the expression of MST2 and YAP1 and that of EMT markers and PRMT1 and 5

In OSCC, the mode of invasion at the invasive front is a histological predictor of recurrence and prognosis (15). An infiltrative growth pattern was shown to be associated with the loss of the epithelial marker E-cadherin and the upregulated expression of vimentin, Slug, and laminin 5, and was considered to be indicative of EMT (22). We previously reported that the expression of PRMT5, a type II arginine methyltransferase, was associated with EMT in OSCC (23). PRMT1, another arginine methyltransferase, is also associated with EMT (24). PRMT1 and PRMT5 have recently been associated with activation of YAP1 activity (10, 11).

**Figure 4:**
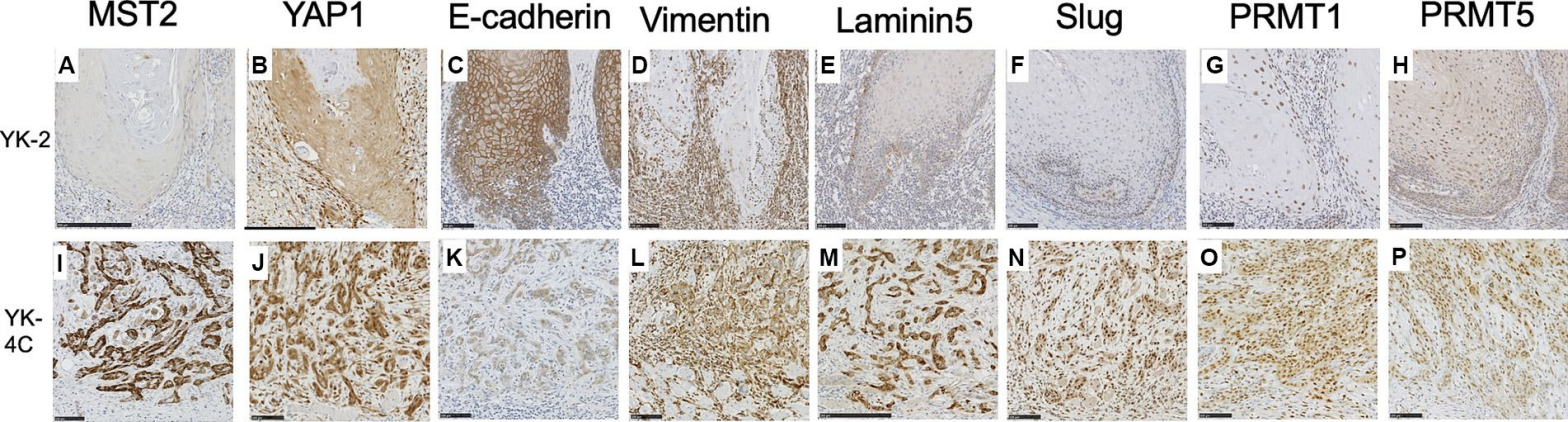
Expression of MST2, YAP1, E-cadherin, vimentin, laminin 5, Slug, PRMT1, and PRMT5 in the invasive front of SCC. Representative cases are shown. **(A)** MST2 expression was low or negative in the majority YK-2 OSCC (28 of 40 YK-1–3 cases). **(B)** Low cytoplasmic expression of YAP1 in most YK-2 OSCC (32 of 40 YK-1–3 cases). **(C)** High membranous expression of E-cadherin in most YK-2 OSCC (36 of 40 YK-1–3 cases). **(D)** Cytoplasmic expression of vimentin in stromal cells of most YK-2 OSCC (31 of 40 YK-1–3 cases). **(E)** Low expression of laminin 5 in YK-2 OSCC (32 of 40 YK-1–3 cases). **(F)** Negative expression of Slug in most YK-2 OSCC (33 of 40 YK-1–3 cases). **(G)** Low nuclear expression of PRMT1 in the majority of YK-2 OSCC (27 of 40 YK-1–3 cases). **(H)** Low expression of PRMT5 in both nuclei and cytoplasm in the majority of YK-2 OSCC (32 of 40 YK-1–3 cases). **(I)** High cytoplasmic expression of MST2 in a subset of YK-4C OSCC (37 of 69 YK-4C, 4D cases). **(J)** High cytoplasmic and nuclear expression of YAP1 in most YK-4C (55 of 69 YK-4C, 4D cases). **(K)** The membranous expression of E-cadherin was typically decreased in YK-4C OSCC compared to YK-2 OSCC (46 of 69 YK-4C, 4D cases vs. 4 of 40 YK-1–3 cases). **(L)** Cytoplasmic expression of vimentin in both cancer and stromal cells in most YK-4C OSCC (55 of 69 YK-4C, 4D cases). **(M)** High cytoplasmic expression of laminin 5 in most YK-4C OSCC (52 of 69 YK-4C, 4D cases). **(N)** Nuclear expression of Slug in most YK-4C OSCC (50 of 69 YK-4C, 4D cases). **(O)** High nuclear and cytoplasmic expression of PRMT1 in the majority of YK-4C OSCC (49 of 69 YK-4C, 4D cases). **(P)** High nuclear and cytoplasmic expression of PRMT5 in in the majority of YK-4C OSCC (56 of 69 YK-4C, 4D cases). Bars **(A–L, N–P)**: 100 μm **(M)**: 250 μm.

Therefore, we stained adjacent sections for E-cadherin, vimentin, Slug, laminin 5, PRMT1, and PRMT5 and investigated whether the expression levels of these molecules and/or those of MST2 and YAP1 were associated with the cancer cell invasion pattern. The results are shown in Figure 4 and Supplementary Table S4.

In the majority of YK-1–3 cases, the cytoplasm of cancer cells was low or negative for MST2 (28 of 40 cases) (Figure 4A) and weakly positive for YAP1 (32 of 40 cases) (Figure 4B). In most YK-1–3 cases (36 of 40 cases), cancer cell membranes retained positivity for E-cadherin (Figure 4C). In most YK-1–3 cases (31 of 40 cases), only stromal cells were positive for vimentin (Figure 4D); only a minority of cases (9 of 40 cases) showed vimentin positivity in both stroma and cancer cells. Similarly, most YK-1–3 cases showed low expression of laminin-5 (Figure 4E, 32 of 40 cases) and Slug (Figure 4F, 33 of 40 cases). The majority of YK-1–3 cases showed low expression of PRMT1 (Figure 4G, 27 of 40 cases) and PRMT5 (Figure 4H, 32 of 40 cases).

In YK-4C, 4D cases, cancer cells more frequently showed high expression of MST2 (Figure 4I, *p* = 0.0169) and YAP1 (Figure 4J, *p* < 0.0001), as compared to YK-1–3 cases. E-cadherin expression was more frequently reduced in the membranes of cancer cells (Figure 4K), as compared to YK-1–3 cases. Cancer cells exhibited high vimentin positivity more frequently than YK-1–3 cases (Figure 4L, *p* < 0.0001). High expressions of Laminin 5 and Slug (Figures 4M,N) were significantly more frequent in YK-4C, 4D cases (laminin-5, 52 of 69 cases; Slug, 50 of 69 cases) than in YK-1–3 cases (laminin-5, 8 of 40 cases; Slug, 7 of 40 cases) (both *p* < 0.0001). Similarly, high expressions of PRMT1 and PRMT5 (Figures 4O,P) were significantly more frequent in YK-4C, 4D cases (PRMT1, 49 of 69 cases; PRMR5, 56 of 69 cases) than in YK-1–3 cases (PRMT1, 13 of 40 cases; PRMT5, 8 of 40 cases) (*p* = 0.0001 for PRMT1, *p* < 0.0001 for PRMT5).

Statistical analyses showed that the reduced expression of E-cadherin and high expression of MST2, vimentin, laminin 5, Slug, PRMT1, and PRMT5 correlated with high nuclear expression of YAP1 (Table 3). High PRMT1 expression was also associated with high PRMT5 expression (Table 4).

**Table 3:** Relationships among YAP1, MST2, EMT markers, and PRMT expression.

		YAP1		
		Low	High	*p*-value
MST2	Low	27	33	**0.0466**
	High	13	36	
E-cadherin	Low	2	41	**<0.0001**
	High	44	22	
Vimentin	Negative	31	14	**<0.0001**
	Positive	15	49	
Laminin 5	Low	35	14	**<0.0001**
	High	11	49	
Slug	Low	21	14	**0.0097**
	High	25	49	
PRMT1	Low	31	16	**<0.0001**
	High	15	47	
PRMT5	Low	38	35	**0.003**
	High	8	28	

The chi-squared test was used to evaluate the associations among YAP1, MST2, EMT markers and PRMT expression. Bold, *p* < 0.05.

**Table 4:** Relationship between the expression of PRMT1 and PRMT5.

		PRMT1		
		Low	High	*p*-value
PRMT5	Low	38	35	**0.0073**
	High	9	27	

The chi-squared test was used to evaluate the associations between PRMT1 expression and PRMT5 expression. Bold, *p* < 0.05.

### Relationships among the expression of YAP1, MST2, PRMT1, and PRMT5 and the prognosis of OSCC

We performed univariate and multivariate Cox proportional hazards analyses to identify parameters affecting the RFS and OS in patients with OSCC.

Univariate analysis revealed that lymphovascular invasion (*p* = 0.0245), neural invasion (*p* = 0.00746), YK-4C, 4D (*p* = 0.0218), high PRMT1 expression (*p* = 0.0282), and high PRMT5 expression (*p* = 0.0287) were correlated with RFS in patients with OSCC. In multivariate analysis, neural invasion, YK-4C, 4D, and high PRMT5 expression correlated with RFS (*p* = 0.0095, 0.013, and 0.0318, respectively) (Table 5).

**Table 5:** Univariate and multivariate analyses of relapse-free survival in patients.

	Univariate analysis		
Factors	HR	95% CI	*p*-value
**Age**			
Under 60	1		
Over 60	0.3323	0.1288–0.8574	0.4247
**Gender**			
Female	1		
Male	0.4783	0.2203–1.0387	0.0623
**Location**			
Tongue	1		
Others	2.2758	0.8279–6.2561	0.1110
**Histological type**			
Grade 1, 2	1		
Grade 3	2.2826	0.9625–5.4135	0.0610
**pT**			
1, 2	1		
3, 4	1.9830	0.9671–4.0662	0.0617
**Stage**			
I, II	1		
III, IV	0.8637	0.1888–3.9519	0.8502
**Ly/v invasion**			
Negative	1		
Positive	2.8591	1.1446–7.1418	**0.0245**
**Neural invasion**			
Negative	1		
Positive	3.1577	1.3579–7.3429	**0.0076**
**LN metastasis**			
Negative	1		
Positive	1.0022	0.2899–3.4644	0.9972
**YK**			
1, 2, 3	1		
4C, 4D	3.0897	1.1782–8.1026	**0.0218**
**YAP1**			
Low	1		
High	1.5948	0.5946–4.2773	0.3538
**MST2**			
Low	1		
High	0.1852	0.0679–0.5054	0.6647
**PRMT1**			
Low	1		
High	1.4202	0.5343–3.7751	**0.0282**
**PRMT5**			
Low	1		
High	2.4479	0.5836–4.5921	**0.0227**

	Multivariate analysis		
Factors	HR	95% CI	*p*-value
**Ly/v invasion**			
Negative	1		
Positive	0.9279	0.4746–1.8143	0.8269
**Neural invasion**			
Negative	1		
Positive	2.4578	1.2455–4.8498	**0.0095**
**YK**			
1, 2, 3	1		
4C, 4D	2.4511	1.2078–4.9743	**0.0130**
**PRMT1**			
Low	1		
High	1.0077	0.4895–2.0745	0.9835
**PRMT5**			
Low	1		
High	1.9402	0.8646–4.3541	**0.0318**

YAP1 RFS. Bold, *p* < 0.05.

Univariate analysis showed that YK-4C, 4D (*p* = 0.003), and high PRMT5 expression (*p* = 0.0389) were correlated with OS in patients with OSCC. In multivariate analysis, only YK-4C and 4D correlated with RFS (*p* = 0.001) (Table 6). The univariate analysis further showed that the MST2 expression was not significantly associated with the OS or RFS in patients with OSCC (Tables 5, 6).

**Table 6:** Univariate and multivariate analyses of overall survival in patients.

	Univariate analysis		
Factors	HR	95 % CI	*p*-value
**Age**			
Under 60	1		
Over 60	3.4683	0.4497–26.7479	0.2328
**Gender**			
Female	1		
Male	2.2317	0.5923–8.4086	0.2356
**Location**			
Tongue	1		
Others	8.1795	0.8802–76.0093	0.0646
**Histological type**			
Grade 1, 2	1		
Grade 3	1.6724	0.2110–13.2540	0.6263
**pT**			
1, 2	1		
3, 4	4.7795	0.7332–31.1573	0.1020
**Stage**			
I, II	1		
III, IV	0.1650	0.0114–2.3774	0.1856
**Ly/v invasion**			
Negative	1		
Positive	0.9390	0.2308–3.8214	0.9300
**Neural invasion**			
Negative	1		
Positive	4.4605	0.7549–26.3542	0.0990
**LN metastasis, *n* (%)**			
Negative	1		
Positive	1.4266	0.1398–14.5635	0.7644
**YK**			
1, 2, 3	1		
4C, 4D	37.6993	3.4172–415.9036	**0.0030**
**YAP1**			
Low	1		
High	0.5632	0.1083–2.9295	0.4949
**MST2**			
Low	1		
High	0.1691	0.0313–0.9131	0.3535
**PRMT1**			
Low	1		
High	0.1552	0.0173–1.3904	0.0958
**PRMT5**			
Low	1		
High	2.4807	0.3639–16.9096	**0.0389**

	Multivariate analysis		
Factors	HR	95 % CI	*p*-value
**YK**			
1, 2, 3	1		
4C, 4D	7.3804	2.1418–25.4317	**0.0015**
**PRMT5**			
Low	1		
High	0.9516	0.3986–2.2720	0.9110

YAP1 OS. Bold, *p* < 0.05.

**Figure 5:**
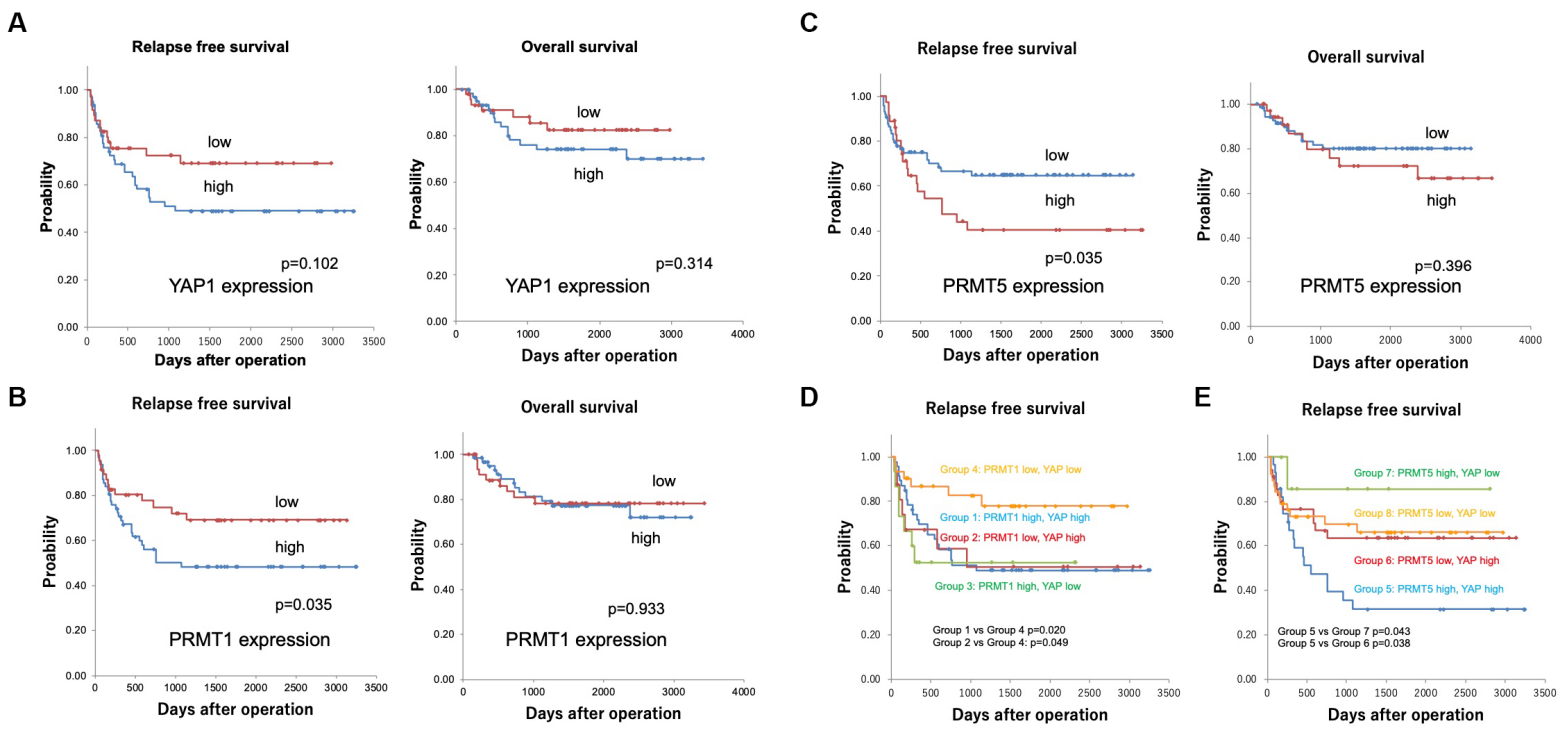
**(A)** Kaplan–Meier curves for the relapse-free (RFS) and overall survival (OS) in patients with YAP1 expression. YAP1 expression was not associated with patient prognosis (5 years survival rate RFS YAP1 low 69.1%, YAP1 high 49.1%, OS YAP1 low 84.8%, YAP1 high 77.7%). **(B)** Kaplan–Meier curves for the relapse-free and overall survival in patients with PRMT1 expression. PRMT1 expression correlated with not OS but RFS (5 years survival rate RFS PRMT1 low 69.0%, PRMT1 high 48.3%, OS PRMT1 low 77.2%, PRMT1 high 78.2%). **(C)** Kaplan–Meier curves for relapse-free survival in patients with PRMT5 expression. PRMT5 expression correlated with not OS but RFS (5 years survival rate RFS PRMT5 low 64.7%, PRMT5 high 40.5%, OS PRMT5 low 80.1%, PRMT5 high 72.1%). **(D)** Kaplan–Meier curves for the relapse-free patient survival according to YAP1 and PRMT1 expression. The RFS was significantly shorter in groups 1 (PRMT1 high and YAP1 high, 5 years survival rate 49.6%) and 2 (PRMT1 low and YAP1 high, 5 years survival rate 50.4%) than in group 4 (PRMT1 low and YAP1 low, 5 years survival rate 77.5%) (*p* = 0.020 and *p* = 0.049, respectively). **(E)** Kaplan–Meier curves for the relapse-free patient survival according to YAP1 and PRMT5 expression. The RFS was significantly shorter in group 5 (PRMT5 high and YAP1 high, 5-year survival rate 31.4%) than in groups 6 (PRMT5 low and YAP1 high, 5 years survival rate 63.6%) and 7 (PRMT5 high and YAP1 low, 5 years survival rate 85.7%) (*p* = 0.038 and 0.043, respectively).

Kaplan–Meier analyses revealed that YAP1 and MST2 expression was not associated with patient prognosis (Figure 5A; Supplementary Figure S1) and that PRMT1 and PRMT5 expression correlated with RFS (both *p* = 0.035) (Figures 5B,C).

Next, we divided the patients into groups according to the expression of YAP1, PRMT1, and PRMT5: group 1, PRMT1 high and YAP1 high; group 2, PRMT1 low and YAP1 high; group 3, PRMT1 high and YAP1 low; group 4, PRMT1 low and YAP1 low; group 5, PRMT5 high and YAP1 high; group 6, PRMT5 low and YAP1 high; group 7, PRMT5 high and YAP1 low; and group 8, PRMT5 low and YAP1 low.

The RFS was significantly shorter in groups 1 and 2 than in group 4 (*p* = 0.020 and *p* = 0.049, respectively) (Figure 5D). RFS was also significantly shorter in group 5 than in groups 6 and 7 (*p* = 0.038 and 0.043, respectively) (Figure 5E).

## Discussion

In this study, we immunohistochemically analyzed the expression of major proteins in the Hippo pathway in OED, CIS, and OSCC. Cytoplasmic expression of MST1, LATS1, and LATS2 was low in OED, CIS, and OSCC. MST2 was highly expressed in the cytoplasm of OED, CIS, and poorly differentiated OSCC but was lost in differentiated OSCC. YAP1 expression was low in well-differentiated OSCC, and high in moderately and poorly differentiated OSCC. An infiltrative invasion pattern was associated with a high expression of MST2 in the cytoplasm and YAP1. High YAP1 expression was associated with loss of E-cadherin and high expression of Slug, vimentin, PRMT1, and PRMT5. Although high YAP1 expression alone did not correlate with OS or RFS, YAP1-high/PRMT1-high and YAP-high/PRMT5-high expression correlated with shorter RFS (both *p* < 0.05). These results suggest that the high co-expression patterns of YAP1/PRMT1 and YAP1/PRMT5 represent aggressive OSCC with EMT.

The kinase cascade forming the core of the Hippo pathway includes MST1/2 and LATS1/2 protein kinases as well as Salvador family WW domain-containing protein 1 as necessary activators of MST1/2 and MOB1 as downstream effectors (5). These core components lead to phosphorylation of the primary downstream effectors YAP1 and TAZ, thereby preventing their translocation to the nucleus (5). We found that MST1, LATS1, and LATS2 were consistently expressed at low levels in the cytoplasm of basal cells of the normal epithelium, dysplastic cells of the OED, and cancer cells. These results suggest that MST1, LATS1, and LATS2 have physiological functions in normal cells and that these functions are retained through the progression from OED to CIS and then to OSCC.

MST1/2 are kinases that regulate many biological processes including apoptosis, cell cycle progression, and migration/invasion (5). The loss of MST1/MST2 has been shown to results in hyperproliferation and tumorigenesis, whereas the overexpression of MST1 prevents tumor growth and proliferation and induces apoptosis (5). Collectively, these findings are consistent with the tumor-suppressive roles of these two kinases.

In contrast, several recent studies have reported a tumor-promoting role of MST2. Park et al. (25) recently reported that overall survival was longer in estrogen receptor (ER)-positive breast cancer patients with low MST2 expression than in those with high MST2 expression using Kaplan–Meier survival analyses, which implies an important interaction between ER and MST2 in breast cancer. Schirmer et al. (26) demonstrated that MST2 is frequently amplified in prostate cancer and that high MST2 expression is associated with a worse prognosis. Pharmacological inhibition of MST2 reduced the growth of prostate cancer cells, indicating its potential of MST2 as a therapeutic target for the treatment of prostate cancer (26). Chen et al. (27) showed that MST2 promotes gastric carcinogenesis by activating the RAS-MAPK signaling pathway and functions as an independent prognostic marker.

In our analyses, the MST2 expression showed a distinctive pattern in OED, CIS, and the different types of OSCC. MST2 was highly expressed in the cytoplasm of OED and CIS cells. In contrast, MST2 expression was lost in a significant proportion of differentiated OSCC, whereas MST2 was highly expressed in all poorly differentiated OSCC. Similarly, the proportion of MST2 high cases were significantly low in SCC with well-defined invasive pattern, as compared to SCC with a diffuse invasion pattern. Although the MST2 expression was not significantly associated with the prognosis in our cohort, a high expression was associated with a poor prognosis in the TCGA data cohort of head and neck cancer (Supplementary Figures S1, S2).

These results suggest that MST2 plays a tumor-suppressive role in a subset of low grade differentiated OSCC and well-defined invasive fronts (YK-1–3), but it may be a tumor-promoting factor in OSCC showing poor differentiation and in a subset of OSCC with an aggressive mode of invasion (YK-4C and 4D). The possibility that MST2 is a tumor-promoting factor in OSCC may be highly speculative, but given the above experimental data (25–27), this possibility may warrant further experimental studies in OSCC.

YAP1 functions as an oncogene; its overexpression inhibits cell contact and promotes cancer cell proliferation and invasion (6). High expression of YAP1 has frequently been reported in various cancers, such as cervical cancer (7), head and neck cancer (7), pancreatic cancer (28), breast cancer (29), intrahepatic cholangiocarcinoma (30), non-small cell lung cancer (31), and liposarcoma (32), and is correlated with poor prognosis, while the loss of YAP1 has potential as a clinical marker in small cell lung cancer (33). Cytoplasmic expression of YAP1 was low in the basal cells of the normal epithelium, dysplastic cells of OED, and cancer cells. These results are largely consistent with previous findings (20) and suggest that YAP1 not only plays a physiological role in basal cells but is also involved in the oncogenesis of OSCC from an early stage.

High/nuclear YAP1 expression was observed in some cases, particularly in those with an advanced pT stage (pT4), an infiltrative invasion pattern (YK-4C or YK-4D), loss of epithelial markers (E-cadherin), and upregulated expression of mesenchymal markers (vimentin, laminin 5, Slug, PRMT1, and PRMT5). Furthermore, high YAP1 expression was associated with worse prognosis. Collectively, these results are somewhat consistent with those of a previous report (8, 34, 35) and suggest that YAP1 is involved in oncogenesis and aggressiveness involving EMT in OSCC. EMT is a complex and reversible biological process, in which an epithelial tumor changes its polar, adhesive phenotype to a mesenchymal phenotype characterized by an increase in cell migration and invasion potential, cytoskeleton remodeling, and resistance to apoptosis (22). In OSCC cell lines, microRNA-27a-3p was found to regulate EMT via the YAP1-OCT4/ Sox2 signal axis (36). In non-small lung cancer, silencing of YAP1 inhibit tumorigenesis and EMT and downregulates Slug expression *in vivo* (37). In colon cancer, upregulated expression of YAP1 was shown to promote EMT and tumor aggressiveness (38). These findings are consistent with those of the present study.

We previously reported that nuclear localization of PRMT1 and PRMT5 is involved in EMT in OSCC (13, 23). PRMTs, including type I (e.g., PRMT1) and type II (e.g., PRMT5), transfer methyl groups to the guanidine nitrogen molecules of arginine residues on their target proteins, and this process affects gene expression, signal transduction, RNA splicing, and other cellular functions (9). PRMT1 has been identified as a positive regulator of YAP1 activity in chondrosarcoma (10). In laryngeal cancer, PRMT5 promotes EMT through YAP1 signaling (11). In the present study, the downregulated expression of E-cadherin and high expression of vimentin, laminin 5, Slug, PRMT1, and PRMT5 correlated with high YAP1 expression. These results are consistent with previous findings on chondrosarcoma and laryngeal cancer and suggest the potential of the YAP1/PRMTs axis as a therapeutic target for the treatment of OSCC.

The present study has several limitations. We were unable to examine the expression of all the proteins involved in the Hippo pathway, including their phosphorylated forms. In addition, changes in the expression do not necessarily indicate changes in the function. YAP target genes may negatively regulate YAP and TAZ; thus, it is possible that the increased expression of MST2 in aggressive OSCC may merely indicate a negative feedback loop caused by an elevated YAP function. Furthermore, the molecular pathways by which MST2, YAP1, and PRMTs contribute to the EMT remain unknown.

During EMT, some cancer cells simultaneously exhibit both mesenchymal and epithelial characteristics, a phenomenon termed partial EMT (pEMT). pEMT is a plastic state in which cells co-express epithelial and mesenchymal markers. In squamous cell carcinoma (SCC), pEMT is regulated via multiple pathways, including the Hippo (YAP being the effector molecule), Notch, and TGF-β pathways and the microenvironment (39).

Cheung et al. (40) showed that the loss of MST1/2 decreased the phosphorylation of MOB1 and YAP1, but did not affect that of LATS1/2, whereas Lu et al. (41) reported that the levels of phosphorylated YAP1 and LATS1/2 were reduced in MST1/2 double knockout livers. However, the precise molecular mechanisms of each Hippo pathway protein interaction in OSCC are yet to be elucidated. Hasegawa et al. (42) showed that YAP1 signaling induces Piezo-type mechanosensitive ion channel component 1 to promote the proliferation of OSCC.

Saladi et al. (43) showed that p63 interacts with co-amplified ACTL6A, encoding an SWI/SNF subunit linked to stem cell and progenitor cell functions. Chromatin remodeling by the ACTL6A/p63 complex suppresses WWC1 to activate YAP1 and promotes tumorigenesis (43). Ahmad et al. (44) showed that DSG3 and YAP activity exhibit mutually exclusive dependence, and desmoglein-3 acts as an important component of the Hippo network in the control of contact inhibition of cell locomotion.

A recent paper proposed that cancer is a multidimensional spatiotemporal “unity of ecology and evolution.” Therefore, the initiation and progression of human cancer can be considered an ecological disease (45). This viewpoint may be helpful for understanding tumor progression from the normal epithelium to OSCC.

In conclusion, a high co-expression pattern of YAP1 and PRMTs indicates aggressive OSCC with EMT at the invasive front. Further studies are needed to elucidate the precise molecular mechanisms by which YAP1 promotes EMT via PRMTs in OSCC.

## Data availability statement

The raw data supporting the conclusions of this article will be made available by the authors, without undue reservation.

## Ethics statement

The studies involving humans were approved by the Ethics Committee of Jichi Medical University. The studies were conducted in accordance with the local legislation and institutional requirements. The ethics committee/institutional review board waived the requirement of written informed consent for participation from the participants or the participants’ legal guardians/next of kin because informed consent was obtained in the form of opt-out on the web-site. Patients who were rejected were excluded.

## Author contributions

YA: conceptualization, investigation, and writing original draft. DM and TY: validated the data. AK: prepared the tissue samples and validated the data. NF, HN, and YM: collected the clinical records. TN: resources, supervision, and writing review and editing. All authors contributed to the article and approved the submitted version.
