# Supplementary material for: The significance of Hippo pathway protein expression in oral squamous cell carcinoma
Source: Front Med (Lausanne). 2024 Feb 20;11:1247625. doi: 10.3389/fmed.2024.1247625 (PMC10912186; doi:10.3389/fmed.2024.1247625)
Supplement: Supplementary file 1 [file Table_1.docx]

| Primary antibody | Clone or code | Type | Source | Dilution | Pretreatment |
| --- | --- | --- | --- | --- | --- |
| MST1 | HPA015270 | rabbit polyclonal | Sigma-Aldrich | 1/300 | autoclaved in 10 mM citrate buffer (pH9.0) at 120ºC for 10 min |
| MST2 | HPA007120 | rabbit polyclonal | Sigma-Aldrich | 1/100 | autoclaved in 10 mM citrate buffer (pH9.0) at 120ºC for 10 min |
| LATS1 | HPA031804 | rabbit polyclonal | Sigma-Aldrich | 1/100 | autoclaved in 10 mM citrate buffer (pH7.0) at 120ºC for 10 min |
| LATS2 | HPA039191 | rabbit polyclonal | Sigma-Aldrich | 1/50 | autoclaved in 10 mM citrate buffer (pH9.0) at 120ºC for 10 min |
| YAP1 | ab52771 | rabbit monoclonal | abcam | 1/100 | autoclaved in 10 mM citrate buffer (pH6.0) at 120ºC for 10 min |
| E-cadherin | clone 36 | mouse monoclonal | BD Biosciences | 1/400 | 10 mM citrate buffer (pH6.0) at 95ºC for 10 min |
| Vimentin | clone V9 | mouse monoclonal | DakoCytomation | 1/400 | incubated in a microwave in 10 mM citrate buffer (pH6.0) at 95ºC for 10 min |
| Slug | clone C19G7 | rabbit monoclonal | Cell signaling | 1/50 | autoclaved in 10 mM citrate buffer (pH7.0) at 120ºC for 10 min |
| Laminin 5 | clone 1-97 | mouse monoclonal | Described in a previous study (46) | 1/50 | autoclaved in 10 mM citrate buffer (pH9.0) at 120ºC for 10 min |
| PRMT1 | SC166963 | rabbit polyclonal | Santacruz | 1/100 | 10% normal horse serum in phosphate-buffered saline |
| PRMT5 | HPA005525 | rabbit polyclonal | Sigma-Aldrich | 1/100 | autoclaved in 10 mM citrate buffer (pH9.0) at 120ºC for 10 min |
| Sup Table 1 Antibodies used for immunohistochemical examination | | | |  |  |
