# Supplementary material for: The significance of Hippo pathway protein expression in oral squamous cell carcinoma
Source: Front Med (Lausanne). 2024 Feb 20;11:1247625. doi: 10.3389/fmed.2024.1247625 (PMC10912186; doi:10.3389/fmed.2024.1247625)
Supplement: Supplementary file 2 [file Table_2.docx]

|  |  | LATS1 | |  |  |  | LATS1 | |  |
| --- | --- | --- | --- | --- | --- | --- | --- | --- | --- |
| Factors | total | low | high | p value | Factors | total | low | high | p value |
| **Age** |  |  |  |  | **Lymphovscular invasion** | |  |  |  |
| Over 60 | 84 | 72 | 12 | 0.2785 | Negative | 43 | 38 | 5 | 0.6017 |
| Under 60 | 46 | 36 | 10 |  | Positive | 66 | 56 | 10 |  |
| **Gender** |  |  |  |  | **Neural invasion** | |  |  |  |
| Male | 70 | 54 | 16 | 0.0513 | Negative | 75 | 62 | 13 | 0.1079 |
| Female | 60 | 54 | 6 |  | Positive | 34 | 32 | 2 |  |
| **Location** |  |  |  |  | **Lymph node metastasis** | |  |  |  |
| Tongue | 95 | 77 | 18 | 0.3105 | Negative | 81 | 70 | 11 | 0.9256 |
| Others | 35 | 31 | 4 |  | Positive | 28 | 24 | 4 |  |
| **Histological type** | |  |  |  | **YK** |  |  |  |  |
| OED | 7 | 5 | 2 | **0.0285*** | 1,2,3 | 40 | 34 | 6 | 0.775 |
| CIS | 14 | 9 | 5 |  | 4C, 4D | 69 | 60 | 9 |  |
| Grade 1,2 | 101 | 89 | 12 | **0.0429**** |  |  |  |  |  |
| Grade 3 | 8 | 5 | 3 |  |  |  |  |  |  |
| **pT** |  |  |  |  |  |  |  |  |  |
| pT1,2,3 | 93 | 80 | 13 | 0.874 |  |  |  |  |  |
| pT4 | 16 | 14 | 2 |  | Age, Gender, and Location including OED and CIS. | | | |  |
| **Stage** |  |  |  |  | *OED, CIS vs Grade 1-3 | |  |  |  |
| I,II | 77 | 66 | 11 | 0.8053 | **Grade 1,2 vs Grade 3 | |  |  |  |
| III, IV | 32 | 28 | 4 |  | The chi-squared test was used to evaluate the associations among LATS1 expression and clinicopathological parameters. Bold, p<0.05. | | | | |
|  |  |  |  |  |  |  |  |  |  |
| Sup Table 2 |  |  |  |  |  |  |  |  |  |
