# Supplementary material for: The significance of Hippo pathway protein expression in oral squamous cell carcinoma
Source: Front Med (Lausanne). 2024 Feb 20;11:1247625. doi: 10.3389/fmed.2024.1247625 (PMC10912186; doi:10.3389/fmed.2024.1247625)
Supplement: Supplementary file 3 [file Table_3.docx]

|  |  | LATS2 | |  |  |  | LATS2 | |  |
| --- | --- | --- | --- | --- | --- | --- | --- | --- | --- |
| Factors | total | low | high | p value | Factors | total | low | high | p value |
| **Age** |  |  |  |  | **Lymphovscular invasion** | |  |  |  |
| Over 60 | 84 | 73 | 11 | 0.7119 | Negative | 43 | 40 | 3 | 0.9067 |
| Under 60 | 46 | 41 | 5 |  | Positive | 66 | 61 | 5 |  |
| **Gender** |  |  |  |  | **Neural invasion** | |  |  |  |
| Male | 70 | 64 | 6 | 0.1613 | Negative | 75 | 70 | 5 | 0.6891 |
| Female | 60 | 50 | 10 |  | Positive | 34 | 31 | 3 |  |
| **Location** |  |  |  |  | **Lymph node metastasis** | |  |  |  |
| Tongue | 95 | 82 | 13 | 0.4312 | Negative | 81 | 73 | 8 | 0.0841 |
| Others | 35 | 32 | 3 |  | Positive | 28 | 28 | 0 |  |
| **Histological type** | |  |  |  | **YK** |  |  |  |  |
| OED | 7 | 4 | 3 | **0.0001*** | 1,2,3 | 40 | 38 | 2 | 0.4758 |
| CIS | 14 | 9 | 5 |  | 4C, 4D | 69 | 63 | 6 |  |
| Grade 1,2 | 101 | 94 | 7 | 0.5609** |  |  |  |  |  |
| Grade 3 | 8 | 7 | 1 |  |  |  |  |  |  |
| **pT** |  |  |  |  |  |  |  |  |  |
| pT1,2,3 | 93 | 85 | 8 | 0.2229 |  |  |  |  |  |
| pT4 | 16 | 16 | 0 |  | Age, Gender, and Location including OED and CIS. | | | |  |
| **Stage** |  |  |  |  | *OED, CIS vs Grade 1-3 | |  |  |  |
| I,II | 77 | 69 | 8 | 0.0502 | **Grade 1,2 vs Grade 3 | |  |  |  |
| III, IV | 32 | 32 | 0 |  | The chi-squared test was used to evaluate the associations among LATS2 expression and clinicopathological parameters. Bold, p<0.05. | | | | |
|  |  |  |  |  |  |  |  |  |  |
| Sup Table 3 |  |  |  |  |  |  |  |  |  |
