# Supplementary material for: The significance of Hippo pathway protein expression in oral squamous cell carcinoma
Source: Front Med (Lausanne). 2024 Feb 20;11:1247625. doi: 10.3389/fmed.2024.1247625 (PMC10912186; doi:10.3389/fmed.2024.1247625)
Supplement: Supplementary file 4 [file Table_4.docx]

|  |  | YK | |  |
| --- | --- | --- | --- | --- |
|  |  | 1~3 | 4C,4D | p value |
| MST2 | low | 28 | 32 | **0.0169** |
|  | high | 12 | 37 |  |
| YAP1 | low | 32 | 14 | **<0.0001** |
|  | high | 8 | 55 |  |
| E-cadherin | decreased | 4 | 46 | **<0.0001** |
|  | retained | 36 | 23 |  |
| Vimentin | stroma | 31 | 14 | **<0.0001** |
|  | stroma and cancer | 9 | 55 |  |
| Laminin 5 | low | 32 | 17 | **<0.0001** |
|  | high | 8 | 52 |  |
| Slug | low | 33 | 19 | **<0.0001** |
|  | high | 7 | 50 |  |
| PRMT1 | low | 27 | 20 | **0.0001** |
|  | high | 13 | 49 |  |
| PRMT5 | low | 32 | 13 | **<0.0001** |
|  | high | 8 | 56 |  |
|  |  |  |  |  |
| Sup. table 4 | The chi-squared test was used to evaluate the associations among YK grade, YAP1, MST2, EMT markers and PRMT expression. Bold, p<0.05. | | | |
|  |  |  |  |  |
|  |  |  |  |  |
