# Supplementary figures and images for: The significance of Hippo pathway protein expression in oral squamous cell carcinoma
Source: Front Med (Lausanne). 2024 Feb 20;11:1247625. doi: 10.3389/fmed.2024.1247625 (PMC10912186; doi:10.3389/fmed.2024.1247625)

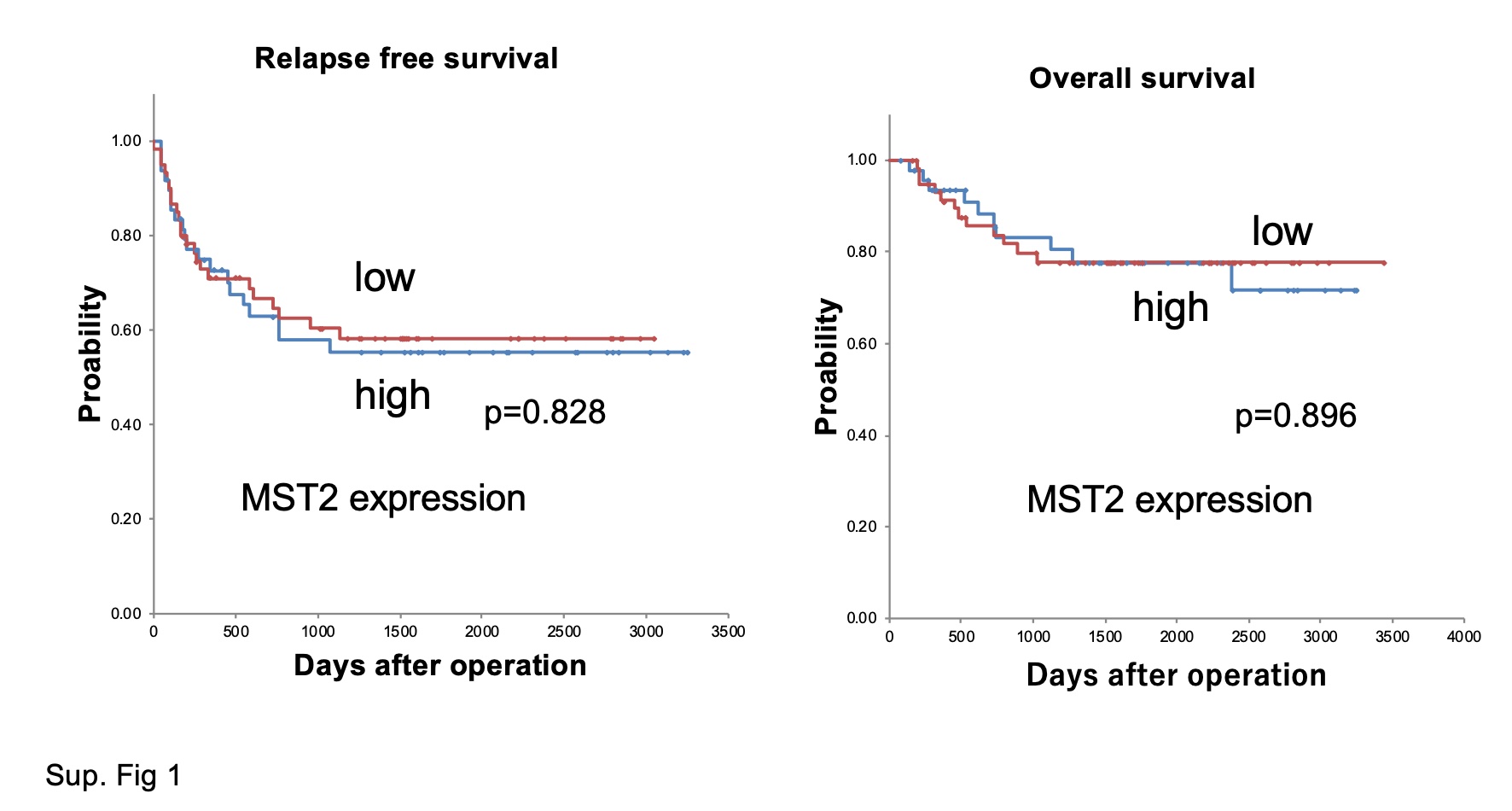

Supplement: Supplementary file 5 [file Image_1.JPEG]

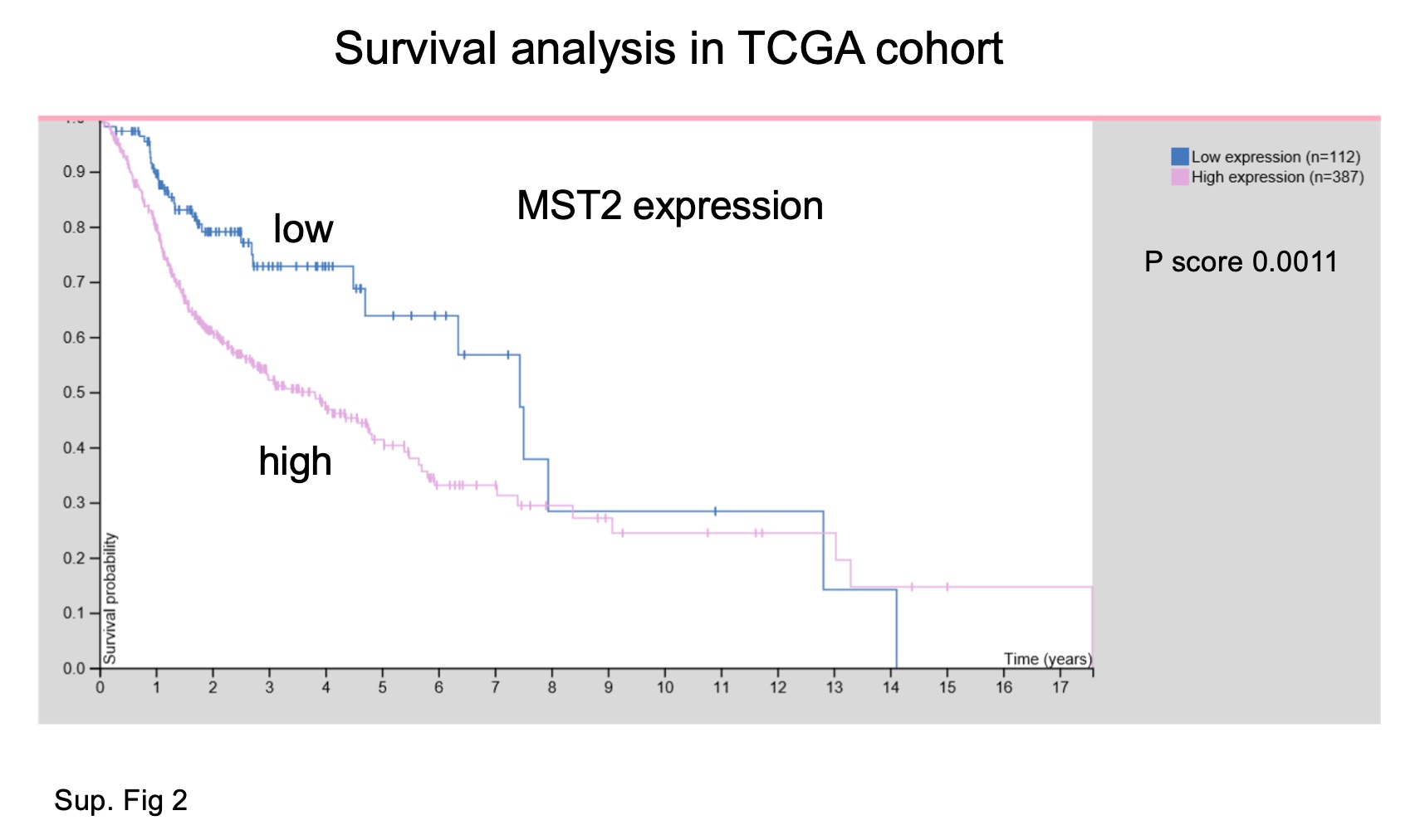

Supplement: Supplementary file 6 [file Image_2.JPEG]
